# Preparation and Characterization of Extruded Yam Starch–Soy Protein Isolate Complexes and Their Effects on the Quality of Dough

**DOI:** 10.3390/foods12020360

**Published:** 2023-01-12

**Authors:** Miaomiao Shi, Xuena Dong, Yanqiu Cheng, Xiaolong Ji, Yanqi Liu, Yizhe Yan

**Affiliations:** College of Food and Bioengineering, Henan Key Laboratory of Cold Chain Food Quality and Safety Control, Food Laboratory of Zhongyuan, Zhengzhou University of Light Industry, Zhengzhou 450002, China

**Keywords:** yam starch, soy protein isolate, extrusion, complexes, dough

## Abstract

Extrusion is a method of processing that changes the physicochemical and rheological properties of starch and protein under specific temperature and pressure conditions. In this study, twin-screw extrusion technology was employed to prepare yam starch–soy protein isolate complexes. The structure and properties of the complexes and their effects on the quality of dough were studied. The results showed changes in the X-ray diffraction, rheology, and in vitro digestibility of the complexes. The extruded starch–protein complex formed an A+V-type crystal structure with the addition of soy protein isolate. A small amount of soy protein isolate could improve the complex’s viscoelasticity. As the content of soy protein isolate increased, the content of slow-digesting starch and resistant starch in the complexes increased, and the digestibility decreased. The microstructure of the dough indicated that the network structure of the puffed yam starch–protein complex dough was more uniform than that of the same amount of puffed yam starch. The moisture distribution of the dough showed that with the addition of extruded flour, the closely bound water content of the dough increased, and the weakly bound water content decreased. The hardness, gumminess, chewiness, and resilience of the dough decreased. In conclusion, extruded starch–protein complexes can improve dough quality and provide technical support for the broad application of yam.

## 1. Introduction

Starch and protein can regulate the function of the body and play very important roles in the body, with great research potential. In addition to their nutritional properties, starch and protein can also be used as gelling agents, thickeners, and stabilizers in food systems. Yam starch is used not only in the field of food but also in the field of medicine. It greatly affects the physicochemical properties of food, such as the texture and rheology [1]. The processing of starch and protein mixtures using special methods (extrusion, high-pressure homogenization, etc.) changes their structural state and, consequently, their original properties. In addition, both the breakdown of starch granules and the denaturation of proteins may lead to further intermolecular and intramolecular interactions between the two polymers [2]. Heat treatment is currently the most common method of forming a starch–protein complex. During the heating process, the structure of starch is destroyed and changed due to water absorption, and the intermolecular hydrogen bonds are also broken. The addition of protein also changes the related indicators of starch gelatinization, retrogradation, rheology, and digestibility. The interaction forces of the starch–protein complex during the cooking process mainly involve hydrogen bonds, electrostatic interactions, and other forces [3]. Moreover, the expansion process of starch granules is restricted by protein. Protein reduces the interaction of amylase with starch and reduces the digestibility of starch [4,5].

The high temperature, high pressure, and high shear force generated in the extrusion process can promote the movement, migration, and extension of the starch molecular chain structure, aggregated structure, and protein molecules. Thus, extrusion has the potential to enhance the interaction between starch and protein molecules [2]. During extrusion, the protein undergoes hydrolysis and denaturation. The higher the protein concentration is, the greater the cross-linking with starch after extrusion will be, which will change the corresponding quality of the product [6]. During extrusion, amylose molecules interact with whey protein to create insoluble polymers, and an increase in the polymer interactions will reduce the water-holding capacity of both starch and protein. As a result, the amount of soluble protein is reduced [7]. The study of changes in the internal structure and physicochemical properties of starch and protein complexes as a result of extrusion could further broaden the avenues of application of starch and protein complexes.

The dough is prepared by mixing flour with water, and different ways of treating the flour can affect the properties of the dough. The stability of dough has been altered by the addition of different varieties of starch or the addition of treated modified starches to wheat dough. For example, dough prepared by replacing part of the wheat flour with gelatinized corn starch had an increased apparent viscosity [8], and the addition of potato starch to wheat dough in moderate amounts significantly improved the stability of the dough [9]. Soy protein isolate also had an effect on the structural properties of dough, with moderate amounts of soy protein isolate resulting in better rheological properties of the dough [10], providing a basis for the development of better-quality wheat bread. In conclusion, partial flour modification, the addition of moderate amounts of soy protein isolate, and the inclusion of bran dietary fiber [11] in the flour were all helpful in obtaining a high-value fortified flour. However, the effect of the interaction between yam starch and soybean isolate protein on the dough during extrusion is challenging, but it is also of broad interest.

In this study, twin-screw extrusion technology was employed to prepare yam starch–soy protein isolate complexes. The structure and properties of the complexes and their effects on the quality of dough were studied. This experiment establishes a foundation for future research on the effects of soybean protein isolate on the properties of yam starch during extrusion processing, encourages the full utilization of yam resources in wheat dough, and provides new ideas for further preparations of bread, noodles, etc.

## 2. Materials and Methods

### 2.1. Materials

Yam starch (YS) was extracted from Chinese yam (Dioscorea opposita Thunb., Jiaozuo, China) in our laboratory at the College of Food and Bioengineering, Zhengzhou University of Light Industry. According to the determination method of Ali et al. [12], the measured protein content of the yam starch was 0.06% and the fat content was 0.12%. Soy protein isolate (SPI) (90.6%, *w*/*w*, dry basis) was purchased from Shanghai Yuanye Biotechnology Co., Ltd. (Shanghai, China). Pancreatin (P7545, 8 × USP) and amyloglucosidase (A7095, 300 U/mL) were obtained from the Sigma Corporation (Ronkonkoma, NY, USA). The glucose oxidase peroxidase (GOPOD) glucose kit was purchased from Megazyme Co., Ltd. (Wicklow, Ireland). All other chemicals used in this study were of analytical grade.

### 2.2. Preparation of Extruded Yam Starch–Soy Protein Isolate (YS-SPI) Complexes

The extruded YS-SPI complexes were prepared using the method of Zhang et al. with minor modifications [13]. Yam starch (YS) was mixed with different amounts of soy protein isolate (SPI) (0, 5, 10, 15, and 20% *w*/*w* of starch). The YS/SPI mixture was extruded using a parallel twin-screw extruder (Process 11, Thermo Fisher Scientific, Waltham, MA, USA) with an 11 mm barrel diameter and 820 mm screw length. The extrusion processing was carried out at 120 °C with the temperatures of the different barrel zones set as 50, 60, 80, 80, 80, 130, and 130 °C. The material moisture content and screw speeds were 30% and 180 rpm, respectively. The extruded product was cut manually and placed in a drying oven at 40 °C for 24 h and then ground and passed through a 149 micro-meter sieve. Based on the added amount of SPI, the extruded YS-SPI complexes were labeled as EYS (control, 0%), SPI-5%, SPI-10%, SPI-15%, and SPI-20%. 

### 2.3. X-ray Diffraction (XRD)

An X-ray diffractogram of the extruded product was obtained using an analytical diffractometer (Bruker D8 Advance, Karlsruhe, Germany). The diffraction patterns were recorded in the range of 5~35° (2θ). The scanning speed was 2°/min and the scanning step was 0.02°. The software Jade 6.0 (Jade Software Corporation Ltd. Inc., Christchurch, New Zealand) was utilized to calculate the degree of relative crystallinity (RC) of the sample [14].

### 2.4. Differential Scanning Calorimetry (DSC)

The endothermic flow curves of the YS-SPI complexes were obtained using a differential scanning calorimeter (Q20, TA instrument Inc., New Castle, DE, USA). Baseline calibration and oven temperature calibration were performed with empty aluminum dishes and indium prior to the experiment. An empty pan was used as the reference. The samples (3 mg, dry basis) and distilled water were added to the aluminum pot using a microliter syringe for a total weight of 12 mg. The samples were equilibrated at room temperature for 12 h and then gradually scanned in a temperate range from 20 °C to 120 °C at a heating rate of 10 °C/min. The resulting data were processed with TA 2000 analysis software.

### 2.5. Dynamic Rheological Measurements

The dynamic rheological behavior of the starch–protein complex was measured using a discovery rheometer (Discovery HR-1, TA instrument Inc., New Castle, DE, USA). The sample was equilibrated at 25 °C for 5 min before measurement. The sample preparation referred to the method of Shi et al. [15], with appropriate modifications, as follows: Place the sample on the flowmeter, remove the excess from the sensor edge, smear the edge with methyl glycerol, perform a frequency sweep in the oscillatory mode, and record the related changes in the statistical G’ and G”. The relevant parameters of the rheometer used were as follows: rotational rheometer: plate-plate; probe diameter: 40 mm; Gap: 1 mm; shear strain: 1%; sweep frequency: 0.1–10.0 Hz; and sweep temperature: 25 °C.

### 2.6. In Vitro Digestibility of Starch

The in vitro digestibility of the samples was determined according to the slightly modified method of Englyst [16]. The sample was accurately weighed to 200 mg (dry weight) in sodium acetate buffer at a concentration of 0.1 mol/L, supplemented with a mixture of porcine trypsin and amyloglucosidase, and shaken to achieve hydrolyzation. The hydrolysis solution was taken at 20 and 120 min, and the enzyme was inactivated by adding ethanol and centrifuged for 10 min. The supernatant was taken and the GOPOD reagent was added, and the color was developed in a water bath for 20 min, while the absorbance was measured at 510 nm. The standard glucose solution and distilled water were also taken for the same treatment and used as the standard and blank control, respectively.

Based on the absorbance of the sample and standard glucose, the contents of fast-digesting starch (RDS), slow-digesting starch (SDS), and resistant starch (RS) were calculated, respectively, and the relevant formulae were as follows:

RDS(%) = (G_20_ − FG) × 0.9

SDS(%) = (G_120_ − G_20_) × 0.9

RS(%) = 1 − (RDS + SDS)

where G_20_ is the glucose content after 20 min of hydrolysis; G_120_ is the glucose content after 120 min of hydrolysis; and FG is the free glucose content of the sample.

### 2.7. Preparation of the Dough

The EYS and SPI-10% were used to replace 10% and 20% (*w*/*w*) of WF to obtain the dough samples. Dough made from natural wheat flour (WF) was used as a control. Based on the substitution quantity of wheat flour, the dough samples were labeled as EYS-10%, EYS-20%, SPI-10%-10%, and SPI-10%-20%.

### 2.8. Scanning Electron Microscopy

The samples were freeze-dried with a freeze dryer (Scientz-10N, Xinzhi Biotechnology Co. Ltd., Ningbo, China) for 1 d and then cut into cubes of approximately 10 × 4 × 5 mm, and the microstructure of the samples was observed using a scanning electron microscope (S4800, Rili Co. Ltd., Hitachi, Tokyo, Japan) at an accelerating voltage of 10 kV. The tested sample was placed on the sample stage with black conductive adhesive, gold-sprayed by vacuum sputtering, observed, and photographed for preservation.

### 2.9. Moisture Distribution Analysis

According to the assay method of Wang [17], implemented with minor additions and modifications, the moisture distribution of the samples was measured with a low-field pulsed nuclear magnetic resonance (LF-NMR) analyzer (NM 120, Niumag Electronics Technology Co. Ltd., Shanghai, China). The operating resonance frequency was 10 MHz. The Carr–Purcell–Meiboom–Gill pulse sequence was selected for the scanning measurements. The experimental parameters were as follows: number of echoes (C0) = 1200, sampling points (TD) = 166398, sampling frequency (SW) = 100.00 kHz, number of repeated scans (NS) = 16, and a half-echo time (TE) = 0.208 ms. The transverse relaxation time (T2) inversion program was used to obtain the moisture distribution spectrum of the dough.

### 2.10. Texture Profile Analysis (TPA)

The textural analysis was performed according to the method of Shi [18], with minor modifications. The abovementioned samples were measured using an XT plus Texture Analyzer (Stable Micro Systems Ltd., Godalming, UK), and the properties of the samples were recorded. The measurement parameters were a P/36R probe with a compression degree of 75%, trigger force of 5 g, and test speed of 4.0 mm/s. The experiment was repeated three times for the determination of this indicator.

### 2.11. Statistical Analysis

All experiments were repeated three times, and the mean and standard deviation were calculated. After sorting and measuring the data using charts, Origin 9.0 software was used for the analysis. Analysis of variance (ANOVA) and post hoc Duncan’s multiple range test (*p* < 0.05) were performed using the SPSS 26.0 statistical software program to determine significant differences between the means for each metric.

## 3. Results and Discussion

### 3.1. XRD 

**Figure 1:**
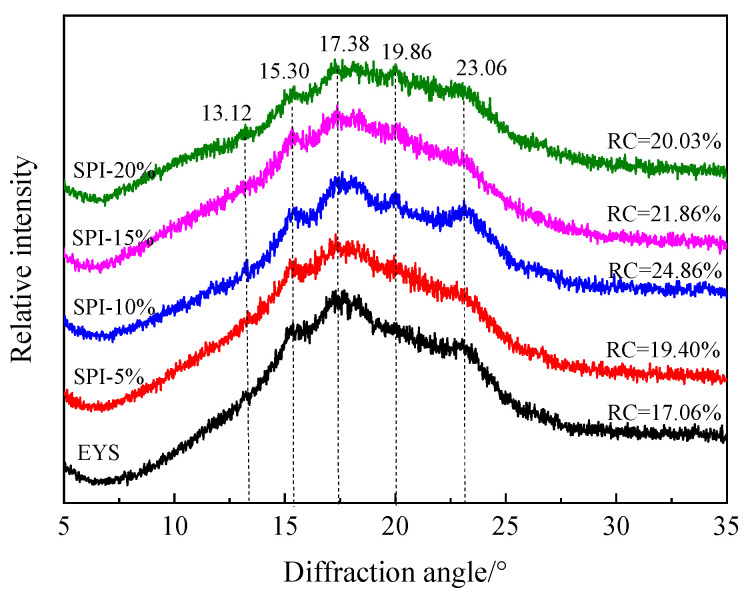
The XRD patterns of extruded starch–protein complex.

The XRD patterns of the extruded yam starch–soy protein isolate complexes are shown in Figure 1. The extruded yam starch had strong diffraction peaks at 15.30°, 17.38°, and 23.06°, which belong to the A-type crystal structure. The X-ray diffraction pattern of the YS-SPI complexes was mainly dominated by broad diffraction peaks, and the characteristic A-type diffraction peaks were weakened. The results indicated that the yam starch was gelatinized, and the soybean protein isolate was denatured. The long-range ordered structure of the yam starch and soy protein isolate was destroyed by extrusion.

With the addition of the soy protein isolate, V-type characteristic peaks appeared at the 2θ of 13.12° and 19.86°. The V-type structure is associated with the formation of complexes between the amylose and lipids or related compounds that occur after starch gelatinization. It was previously reported that V-type crystals reduced the digestibility of carbohydrates, thereby reducing the blood sugar and insulin response [19]. With the increase in the SPI content, the V-type diffraction peaks of the YS-SPI complexes at 13.12° and 19.86° were enhanced to a certain extent. The relative crystallinity first increased and then decreased with the addition of soy protein isolate, but it was still higher than that of the control group. This showed that the increase in the soy protein isolate had a certain promoting effect on the V-type crystallization.

### 3.2. DSC

The gelatinization parameters of the YS-SPI complexes are summarized in Table 1. The peak temperature (T_p1_) of the yam starch changed slightly with the addition of the soy protein isolate. It may be that the integrity of the starch granules was more severely disrupted by the addition of soybean isolate and extrusion, and the interaction between the amylose and amylose or amylopectin was weakened, which made the yam starch easier to paste. Compared with the extruded yam starch, the gelatinization enthalpy value (ΔH_1_) of the starch in the extruded complexes decreased with the increase in the protein addition ratio. When 20% soy protein isolate was added, the ΔH_1_ decreased from 1.87 J/g to 1.26 J/g. The presence of protein reduces the interaction between starch and water in the complex, because it affects the water mobility of starch granules during gelatinization [5]. Therefore, the gelatinization enthalpy decreased. In addition, the reduction in the gelatinization enthalpy may be due to a reduction in the relative concentration of starch in the complexes. The double helix structure of the complexes was reduced, enabling a small amount of heat to unwind the double helix during the gelatinization process [20]. The increase in the additional amount of soy protein isolate reduced the amount of starch-endogenous lipid complexes, which may have resulted in a decreasing trend of the ΔH_2_ value.

### 3.3. Rheological Properties

**Figure 2:**
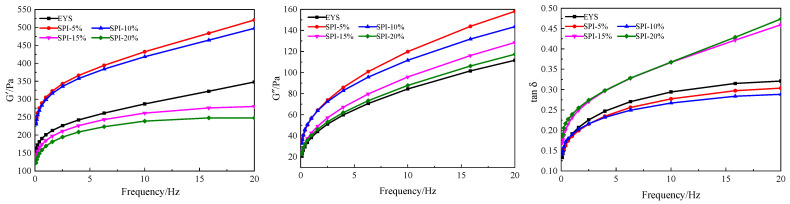
The rheological properties of the extruded YS and YS-SPI complexes.

The dynamic rheological curves of the yam starch with different amounts of soy protein isolate are shown in Figure 2. The storage modulus (G’) of the samples in the frequency range of 0–20 Hz was significantly higher than the loss modulus (G”), and there was no crossover. This indicated that the soy protein isolate and yam starch composite gel were typical weak gel dynamic rheology systems [21]. This result was similar to that of the research of Chen et al. [22]. The G’ and G” values of all the sample pastes increased steadily with the frequency, indicating the formation of a weak gel behavior [23].

As can be seen in Figure 2, the storage modulus of the extruded YS-SPI complexes increased with the lower SPI additions (5% and 10%). This may be because the small amount of soybean protein isolate added before the extrusion treatment changed its original properties and affected the original rheological properties. The storage modulus of the complex gel decreased with the addition of large amounts of SPI (15% and 20%). This may be due to the addition of soy protein isolate molecules surrounding or attached to the surface of the starch granules, which hindered the exudation of the amylose molecules and the swelling of the starch granules [24]. The G’ and G” curves of the system were decreased in an addition-dependent manner with the addition of SPI. In other words, the viscoelasticity of the sample decreased with the increase in SPI at the same frequency. Upon its addition, the soy protein isolate surrounded the surface of the starch particles, preventing the water absorption and expansion of starch granules and forming a crosslinking network of amylose molecules. Therefore, the system’s G’ and G” were reduced in a concentration-dependent manner. Sarabhai et al. [25] also came to similar conclusions when they studied the effect of protein on starch rheological behavior.

The tan δ value is the ratio of G” and G’. The larger the value of tan δ is, the stronger the system’s fluidity is. The smaller the value of tan δ is, the stronger the solid performance is. The presence of SPI increased the tan δ value of the complex, indicating a reduction in the solid-like behavior of the system. With the increase in the soy protein isolate concentration, the viscoelastic behavior transformed from a solid-like state into a liquid-like state [5].

### 3.4. In Vitro Starch Digestibility

The digestibility of the samples was calculated by measuring the glucose released during starch hydrolysis. Studies have shown that the digestion degree and speed of starch and starch-containing foods are related to the release of glucose, which is represented by the contents of RDS, SDS, and RS in the starch system [26].

**Figure 3:**
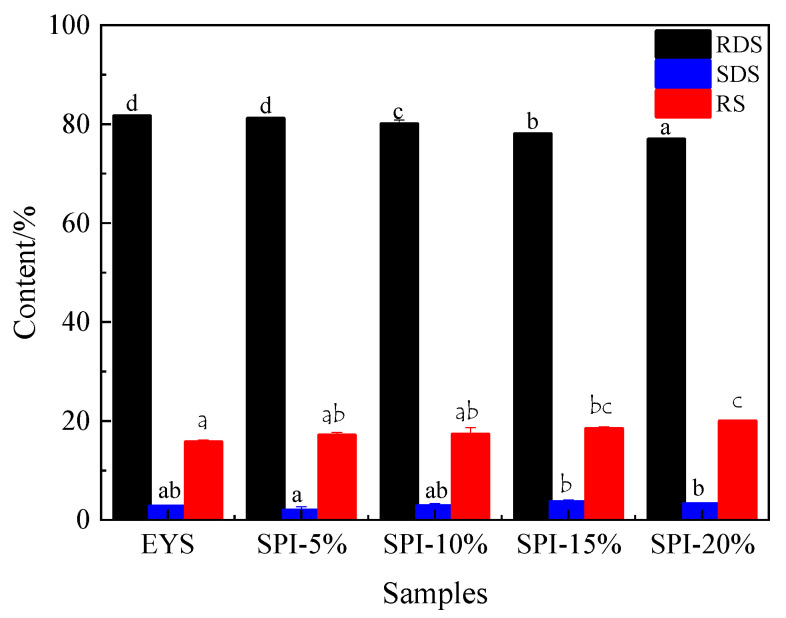
In vitro digestion of the extruded YS and YS-SPI complexes. Different small letter in the same items indicate significant differences (*p* < 0.05).

As shown in Figure 3, there was a significant difference in the digestibility of the extruded starch samples (*p* > 0.05). Compared with the extruded YS, the SDS and RS contents of the extruded YS-SPI complexes were significantly increased, and the RDS content was significantly reduced. When the content of soy protein isolate was 20%, the RDS content decreased to 76.86%, and the RS content increased to 19.93%. This indicated that the digestibility of the complex gradually reduced with the increase in the proportion of soy protein isolate. This may be related to the protein matrix enwrapping the starch networks and restricting hydrolysis. The proteins were enwrapped and surrounded by starch granules, which may have been a barrier to the delay of enzymatic hydrolysis [27]. Ezeogu et al. [28] found that sorghum and maize endosperm protein matrices had lower starch digestibility. This was related to the barriers of starch gelatinization and starch hydrolysis, caused by the enwrapping of the starch granules with a complete protein matrix. Choi, Woo, Ko, and Moon [29] also found similar results. They used a confocal laser scanning microscope to examine the microstructure of waxy sorghum flour. They found that the native protein in the waxy sorghum flour formed a network on the starch granules. This significantly reduced the starch degradation caused by α-amylase and amyloglucosidase. Therefore, soy protein isolate had significant effects on the digestive properties of the extruded YS-SPI complexes, and the performance of the complexes could be improved by adjusting the content of soy protein isolate.

### 3.5. Microstructure Analysis of Dough

**Figure 4:**
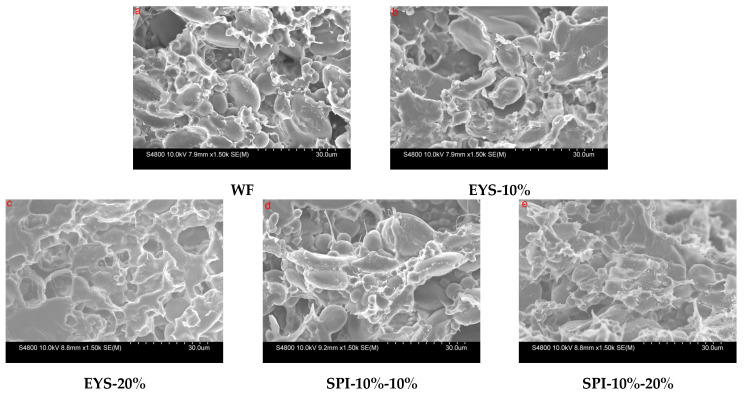
Effects of different replacement amounts of extruded flour on the microstructure of dough (×1500).

Figure 4 shows the microstructure of the freeze-dried dough with extruded flour. As the amount of extruded flour increased, the dough showed different microstructures. In the control dough, a continuous transparent sheet gluten network structure was formed, and the network was embedded with intact starch and other granule molecules that were clearly visible. On the one hand, when the extruded flour was added, the continuity of the network structure formed by the gluten protein became worse, and the degree of protein weakening in the dough increased. On the other hand, the gelatinized extruded flour was sticky after absorbing water and could be attached to the network structure of the wheat protein. A network structure different from that of gluten protein was formed. When the substitute amount increased, the dough exhibited pores similar to the network structure state. During the freeze-drying process, the dough acquires many small pores due to the formation and sublimation of ice crystals [30,31], which may be directly related to the properties of the gelatinized starch. This is directly related to the gelatinizing properties of starch.

Under the same conditions, the network structure of the dough with the same amount of extruded YS-SPI complex was more uniform than that with extruded YS. During extrusion, the high temperature caused the irreversible denaturation of the soy protein isolate. Under conditions of adequate moisture, the increase in the temperature led to the expansion of the protein structure and the exposure of hydrophobic groups. During subsequent aggregation, the denatured proteins were stabilized by hydrophobic interactions and disulfide bond formation [32].

### 3.6. Moisture Distribution of the Dough

The distribution of moisture in the dough was measured using low-field NMR. The parameters related to the moisture distribution of the dough are shown in Table 2. Moisture migration between starch and lipids, proteins, and other non-starch components during dough kneading could be better understood. It is helpful to determine the applicable conditions of the dough. It is generally understood that T represents the relaxation time of the water in each state, and A represents the ratio of the peak area of the water in each state with respect to the total area. T_21_ (0.01~1.00 ms) represents the bound water closely bound to starch and gluten proteins. T_22_ (1.00~50.00 ms) represents the weakly bound water bound to macromolecules such as starch and protein. T_23_ (50.00~200.00 ms) represents free water. A_21_ represents the peak area percentage of the bound water. A_22_ represents the peak area percentage of the weakly bound water. A_23_ represents the peak area percentage of the free water [33,34].

The proportions of the three states of the water in the dough were as follows: A_22_ (weakly bound water) > A_21_ (bound water) > A_23_ (free water). With the addition of extruded flour, the proportion of closely bound water A_21_ increased significantly, and the proportion of weakly bound water A_22_ decreased. The higher the ratio of A_21_ is, the higher the bound water content of the dough will be. As a result, the better the dough’s water-holding capacity is, the more stable the dough’s structure will be [35]. The free water A_23_ decreased as the additional amount increased, and the dough’s fluidity weakened. This showed that the extruded flour’s hydrophilic effect reduced the amount of free water in the dough and increased the ability of the water molecules to be bound. At this time, the T_21_ of the bound water in the dough due to its combination with the extruded flour was correspondingly increased. At the same substitution amount, the free water in the dough with the extruded starch–protein complex added was lower than that to which extruded yam flour and starch were added. This can be related to starch–protein interactions during extrusion. The results indicated that the gelatinized starch and denatured protein in extruded flour have sufficient water binding sites and can also affect the movement of water molecules. Thereby, the moisture distribution of the dough was promoted.

### 3.7. Texture Profile Analysis (TPA)

The texture analysis of dough is an indispensable theoretical method for the study of dough. The internal tissue state of the dough can be objectively analyzed using a texture analyzer. According to Table 3, the dough with extruded flour had a lower hardness, viscosity, and chewiness than the control dough. When the amount of extruded flour was increased from 0% to 20%, the hardness of the dough decreased from 390.6 N to 139.8 N, and the chewiness decreased from 113.1 N to 15.2 N. This may be due to the absence of gluten in the extruded starch–protein complex, and the substitution of extruded flour resulted in a decrease in the gluten content of the dough, which in turn decreased the hardness and chewiness of the dough [36]. Zhang [37] found that the addition of extruded mung bean powder significantly decreased the firmness and chewiness of composite noodles, which is similar to the results of this study. The springiness and gumminess ranged from 0.23 to 0.78 and 59.6 N to 181.2 N, respectively. The addition of extruded yam starch significantly reduced the viscosity and elasticity of the dough, and this may lead to difficulty in the formation of an ordered starch network structure in the dough and reduce the hardness and springiness. It has been reported that the viscosity and elasticity of extruded millet flour–wheat composite bread decreased significantly with the increase in the extruded millet flour content [38]. However, the springiness of the dough increased after the extruded yam starch–soy protein isolate complex was added, which was directly related to the starch–protein interaction during the extrusion process.

Under the same conditions, the dough with extruded YS-SPI complexes had a higher hardness, springiness, gumminess, and chewiness than the dough with extruded starch. This may be due to the attachment of the soy protein isolate molecules to the surface of the starch granules, which affected the water absorption and expansion of the starch granules, and the exudation of amylose molecules. The resilience of the dough decreased with the addition of the extrusion flour. Additionally, it decreased with the increase in the replacement amount of extruded flour. A study conducted by Abdelghafor et al. [39] showed that when whole sorghum flour was added to wheat flour, the wheat flour gluten was diluted. Therefore, the recoverability of the noodles decreased with the increase in the whole sorghum flour content. This is consistent with the results of this study. The dough samples with the extruded flour texture parameter displayed a slight tendency to lower when the substitution increased, and this slight tendency was more evident when the degree of substitution increased to 20%.

## 4. Conclusions

In this paper, the structure and physicochemical properties of the extruded YS-SPI complexes and their effects on dough quality were investigated. The extruded YS-SPI complexes formed an A+V-type crystal structure, and the starch digestibility was reduced. The low content of soy protein isolate could also improve the viscoelasticity of the complex. The tightly bound water content of the dough increased. The fluidity of the dough was weakened. Under the same conditions, the hardness, springiness, gumminess, and chewiness of the dough with the extruded YS-SPI complexes were higher than those of the extruded YS. In conclusion, the extruded YS-SPI complexes not only added nutrients to the food but also improved the quality of the dough. Therefore, dough can be modified by adding extruded flour, providing a new green and pollution-free technical idea for the production of fried dough sticks, dried noodles, bread, and other products.

## Figures and Tables

**Table 1:** The thermal properties of extruded YS and YS-SPI complexes.

Samples	To_1_ (°C)	Tp_1_ (°C)	Tc_1_ (°C)	ΔH_1_ (J/g)	To_2_ (°C)	Tp_2_ (°C)	Tc_2_ (°C)	ΔH_2_ (J/g)
EYS	51.44 ± 0.36 ^b^	62.36 ± 0.54 ^ab^	74.66 ± 0.16 ^a^	1.87 ± 0.07 ^a^	85.42 ± 0.45 ^b^	91.25 ± 0.30 ^b^	99.08 ± 0.42 ^b^	0.54 ± 0.08 ^b^
SPI-5%	50.28 ± 0.16 ^a^	63.01 ± 0.21 ^b^	74.54 ± 0.71 ^a^	1.69 ± 0.36 ^a^	85.36 ± 0.38 ^b^	91.27 ± 0.44 ^b^	98.75 ± 0.98 ^b^	0.40 ± 0.05 ^ab^
SPI-10%	51.02 ± 0.26 ^b^	62.31 ± 0.22 ^ab^	74.73 ± 0.50 ^a^	1.68 ± 0.18 ^a^	83.26 ± 0.36 ^a^	89.70 ± 0.30 ^a^	94.99 ± 0.12 ^a^	0.71 ± 0.03 ^c^
SPI-15%	50.38 ± 0.19 ^a^	61.70 ± 0.97 ^a^	74.41 ± 0.24 ^a^	1.51 ± 0.65 ^ab^	85.20 ± 0.83 ^b^	90.86 ± 0.54 ^b^	99.39 ± 0.24 ^b^	0.38 ± 0.12 ^a^
SPI-20%	51.31 ± 0.55 ^b^	61.32 ± 0.40 ^a^	73.67 ± 0.80 ^a^	1.26 ± 0.12 ^b^	84.86 ± 0.28 ^b^	90.64 ± 0.67 ^b^	98.36 ± 1.02 ^b^	0.34 ± 0.11 ^ab^

Values are expressed as means ± standard deviation of three measurements. Means with different lowercase letters in the same column indicate significant differences (*p* < 0.05). EYS indicates extruded yam starch; SPI-5% indicates the addition of SPI to the extruded YS-SPI complex at an amount of 5%; SPI-10% indicates the addition of SPI to the extruded YS-SPI complex at an amount of 10%; SPI-15% indicates the addition of SPI to the extruded YS-SPI complex at an amount of 15%; SPI-20% indicates the addition of SPI to the extruded YS-SPI complex at an amount of 20%; To_1_/To_2_, onset temperature; Tp_1_/Tp_2_, peak temperature; Tc_1_/Tc_2_, conclusion temperature; ΔH_1_/ΔH_2_, gelatinization enthalpy.

**Table 2:** Effects of different replacement amounts of extruded flour on the relaxation time and water quantity of dough.

Samples	T_21_ (ms)	T_22_ (ms)	T_23_ (ms)	A_21_ (%)	A_22_ (%)	A_23_ (%)
WF	0.56 ± 0.05 ^ab^	9.13 ± 0.94 ^a^	137.10 ± 0.00 ^a^	12.39 ± 0.34 ^a^	86.85 ± 0.37 ^c^	0.74 ± 0.02 ^a^
EYS-10%	0.48 ± 0.05 ^a^	11.35 ± 0.00 ^b^	147.92 ± 15.30 ^ab^	14.18 ± 0.12 ^c^	84.89 ± 0.12 ^a^	0.92 ± 0.00 ^c^
EYS-20%	0.87 ± 0.09 ^c^	13.14 ± 0.00 ^c^	158.74 ± 0.00 ^ab^	13.73 ± 0.10b ^c^	85.47 ± 0.12 ^ab^	0.79 ± 0.02 ^ab^
SPI-10%-10%	0.48 ± 0.05 ^a^	11.35 ± 0.00 ^b^	158.74 ± 0.00 ^ab^	13.70 ± 0.09 ^bc^	85.48 ± 0.15 ^ab^	0.91 ± 0.06 ^c^
SPI-10%-20%	0.73 ± 0.29 ^ab^	13.14 ± 0.00 ^c^	171.26 ± 17.71 ^b^	13.18 ± 0.56 ^ab^	85.93 ± 0.62 ^b^	0.88 ± 0.05 ^bc^

Values are expressed as means ± standard deviation of three measurements. Means with different lowercase letters in the same column indicate significant differences (*p* < 0.05). WF indicates dough made from natural wheat flour; EYS-10% indicates dough samples made with extruded yam starch instead of 10% (*w*/*w*) wheat flour; EYS-20% indicates dough samples made with extruded yam starch instead of 20% (*w*/*w*) wheat flour; SPI-10%-10% indicates dough samples made with SPI-10% instead of 10% (*w*/*w*) wheat flour; SPI-10%-20% indicates dough samples made with SPI-10% instead of 20% (*w*/*w*) wheat flour. T_21_ represents bound water closely bound to starch and gluten proteins. T_22_ represents weakly bound water bound to macromolecules such as starch and protein. T_23_ represents free water. A_21_ represents the peak area percentage of bound water. A_22_ represents the peak area percentage of weakly bound water. A_23_ represents the peak area percentage of free water.

**Table 3:** Effects of different replacement amounts of extruded flour on the texture of the dough.

Samples	Hardness (N)	Springiness	Gumminess (N)	Chewiness (N)	Resilience
WF	390.69 ± 14.82 ^d^	0.62 ± 0.10 ^b^	181.17 ± 9.23 ^d^	113.10 ± 24.82 ^c^	0.37 ± 0.04 ^c^
EYS-10%	193.95 ± 8.47 ^b^	0.25 ± 0.01 ^a^	80.02 ± 7.08 ^b^	18.62 ± 3.01 ^a^	0.28 ± 0.00 ^b^
EYS-20%	139.83 ± 5.11 ^a^	0.23 ± 0.00 ^a^	59.55 ± 1.00 ^a^	15.20 ± 0.61 ^a^	0.15 ± 0.00 ^a^
SPI-10%-10%	247.43 ± 1.23 ^c^	0.78 ± 0.01 ^c^	100.77 ± 0.71 ^c^	78.81 ± 1.90 ^b^	0.25 ± 0.00 ^b^
SPI-10%-20%	141.43 ± 6.29 ^a^	0.76 ± 0.00 ^c^	63.61 ± 0.71 ^a^	61.08 ± 0.47 ^b^	0.17 ± 0.00 ^a^

Values are expressed as means ± standard deviation of three measurements. Means with different lowercase letters in the same column indicate significant differences (*p* < 0.05). WF indicates dough made from natural wheat flour; EYS-10% indicates dough samples made with extruded yam starch instead of 10% (*w*/*w*) wheat flour; EYS-20% indicates dough samples made with extruded yam starch instead of 20% (*w*/*w*) wheat flour; SPI-10%-10% indicates dough samples made with SPI-10% instead of 10% (*w*/*w*) wheat flour; SPI-10%-20% indicates dough samples made with SPI-10% instead of 20% (*w*/*w*) wheat flour.

## Data Availability

The data from this study are not publicly available at this time but can be obtained from the first author upon a reasonable request.
